# The Influence of the Composition of Ru_100−*x*_Al_*x*_ (*x* = 50, 55, 60, 67) Thin Films on Their Thermal Stability

**DOI:** 10.3390/ma10030277

**Published:** 2017-03-10

**Authors:** Marietta Seifert, Gayatri K. Rane, Steffen Oswald, Siegfried B. Menzel, Thomas Gemming

**Affiliations:** IFW Dresden, SAWLab Saxony, P.O. Box 270116, 01171 Dresden, Germany; g.k.rane@ifw-dresden.de (G.K.R.); s.oswald@ifw-dresden.de (S.O.); s.menzel@ifw-dresden.de (S.B.M.); t.gemming@ifw-dresden.de (T.G.)

**Keywords:** RuAl thin film, high-temperature stability, surface acoustic waves

## Abstract

RuAl thin films possess a high potential as a high temperature stable metallization for surface acoustic wave devices. During the annealing process of the Ru-Al films, Al${}_{2}$O${}_{3}$ is formed at the surface of the films even under high vacuum conditions, so that the composition of a deposited Ru${}_{50}$Al${}_{50}$ film is shifted to a Ru-rich alloy. To compensate for this effect, the Al content is systematically increased during the deposition of the Ru-Al films. Three Al-rich alloys—Ru${}_{45}$Al${}_{55}$, Ru${}_{40}$Al${}_{60}$ and Ru${}_{33}$Al${}_{67}$—were analyzed concerning their behavior after high temperature treatment under high vacuum and air conditions in comparison to the initial Ru${}_{50}$Al${}_{50}$ sample. Although the films’ cross sections show a more homogeneous structure in the case of the Al-rich films, the RuAl phase formation is reduced with increasing Al content.

## 1. Introduction

High temperature stable metallizations attract attention for a wide field of applications, especially in the area of high temperature stable sensors and, to a minor degree, also as electrodes for the high temperature characterization of acoustic properties of piezoelectric crystals. Recently, various metals, alloys and compounds have been characterized and optimized concerning their high temperature (up to 1000 ${}^{\circ }$C) applicability [1,2,3,4]. One of the materials described as promising for high temperature applications up to 800 ${}^{\circ }$C under high vacuum conditions is the RuAl alloy [5,6,7,8]. However, during its annealing at high temperature, Al gets oxidized even under high vacuum conditions and a 15–20 nm thick Al${}_{2}$O${}_{3}$ layer is formed at the sample surface (annealing temperature: 800 ${}^{\circ }$C, annealing time: 10 h) [7,8,9]. Due to the formation of the Al${}_{2}$O${}_{3}$, the overall content of Al in the film after the annealing is reduced as compared to the as-deposited state. This means that if a stoichiometric Ru${}_{50}$Al${}_{50}$ film is deposited, after the annealing, the composition is shifted to an Ru-rich alloy. From former investigations of 110 nm thick Ru${}_{50}$Al${}_{50}$ films on Si/SiO${}_{2}$ substrates, which where annealed at 800 ${}^{\circ }$C for 10 h under high vacuum conditions, we estimate that the formed Al${}_{2}$O${}_{3}$ cover layer contains up to about 20 at % of the Al of the initially deposited Ru${}_{50}$Al${}_{50}$ film.

Investigation of the oxidation behavior of thicker RuAl films (200–600 nm) [10,11] as well as of bulk samples [12,13,14] reveal a complicated, partly multilayered oxide scale on top of the samples consisting of alternating Al${}_{2}$O${}_{3}$ and Ru layers.

On the other hand, dense Al${}_{2}$O${}_{3}$ cover layers are known to act as passivation layers, so that the formation of an Al${}_{2}$O${}_{3}$ thin layer at the film surface might also improve the long-term stability of the subjacent film. One way to compensate for the lack in Al due to some formation of Al${}_{2}$O${}_{3}$ is to deposit the Ru-Al alloy with increased Al content. This paper reports on the effects of the increased Al content on the RuAl phase formation and high temperature stability of Ru${}_{100-x}$Al${}_{x}$ (x=50,55,60,67) thin films.

## 2. Experimental Section

Ru-Al thin films with a constant thickness of 110 nm in the as-deposited state have been deposited by magnetron co-sputtering from elemental Ru (purity: 99.95%) and Al (purity 99.9995%) targets on thermally oxidized Si substrates (Si/SiO${}_{2}$ (for details of deposition, see [7])). Besides the preparation of the stoichiometric Ru${}_{50}$Al${}_{50}$ phase, samples with a higher content of Al were prepared—namely, Ru${}_{100-x}$Al${}_{x}$ with x=55,60,67. The composition was changed by increasing the sputtering power of Al by 20%, 50% and 100%, resulting in the above described compositions in the as-deposited state.

After preparation, the obtained atomic compositions were checked with glow discharge optical emission spectroscopy (GDOES, GDA 750 HR, Spectruma Analytik GmbH, Hof, Germany). The instrument was calibrated using two different RuAl bulk standards in combination with energy dispersive X-ray spectroscopy (EDX) in a scanning electron microscope (SEM, Zeiss Ultra Plus, Carl Zeiss Microscopy GmbH, Jena, Germany). Applying the determined calibration, the measurements of the various co-sputtered thin films showed that the Ru-content in the as-deposited films was systematically 5 at % higher than intended; a deviation which is, in this case, within the measurement precision of the applied methods, so that the annotation is kept as above mentioned. In the following, the composition Ru${}_{100-x}$Al${}_{x}$ (x=50,55,60,67) always refers to the as-deposited state.

After deposition, the samples were annealed at 600 ${}^{\circ }$C (air), 800 ${}^{\circ }$C (air and high vacuum) or 900 ${}^{\circ }$C (air and high vacuum) for 10 h. During the treatment under high vacuum conditions, the pressure in the oven was below 10${}^{-5}$ mbar.

The phase formation before and after the heat treatment was investigated by X-ray diffraction (XRD, Philips X’Pert PW3040/00, Co-Kα, PANalytical, Almelo, The Netherlands) in Bragg Brentano geometry. The surface morphology was imaged by SEM. Cross sections of the layer stacks have been prepared by the focussed ion beam technique (FIB, Zeiss 1540 XB CrossBeam, Carl Zeiss Microscopy GmbH) and have been imaged by SEM.

The global chemical composition was analyzed by Auger electron spectroscopy (AES, JEOL JAMP-9500F Field Emission Auger Microprobe, JEOL (Germany) GmbH, Freising, Germany). The measurement of depth profiles was realized by sputtering with Ar ions for 60 s with an ion beam at 1 keV and a current of $0.7\times 10^{-6}$ A (corresponding to a milling rate of 6.6 nm/min for amorphous SiO${}_{2}$). For some samples, it was necessary to increase the ion energy to 2 keV (corresponding to a milling rate of 15–20 nm/min of SiO${}_{2}$). In the following, this is denoted for the respective samples. Concentration quantification was done using single element standard sensitivity factors [15]. Additionally preferential sputtering during ion bombardement can alter the surface concentration; thus, the absolute measured concentrations may differ from the bulk ones. However, relative concentration changes after thermal treatment can always be followed. The sputtered area is about 1 mm${}^{2}$ and the measurement region for the AES within this is about 15×15 μm${}^{2}$.

Measurements of the electrical resistance were carried out by using the van der Pauw method [16] for thin films. Several measurements were done with constant current of 5 and 10 mA and alternating direction of the electric current using a constant current source and a nanovoltmeter (2182A–Nanovoltmeter, KEITHLEY-TEKTRONIX, Inc., Beaverton, OR, USA) for voltage measurement.

The analysis of the microstructure was performed by high-angle annular dark field scanning transmission electron microscopy (HAADF-STEM, Technai F30, FEI company, Hillsboro, OR, USA). The image contrast is sensitive to the chemical composition. The local chemical composition was revealed by energy dispersive X-ray spectroscopy (EDX, EDAX Company, Mahwah, NJ, USA) in the same instrument.

## 3. Results

### 3.1. Phase Formation

**Figure 1:**
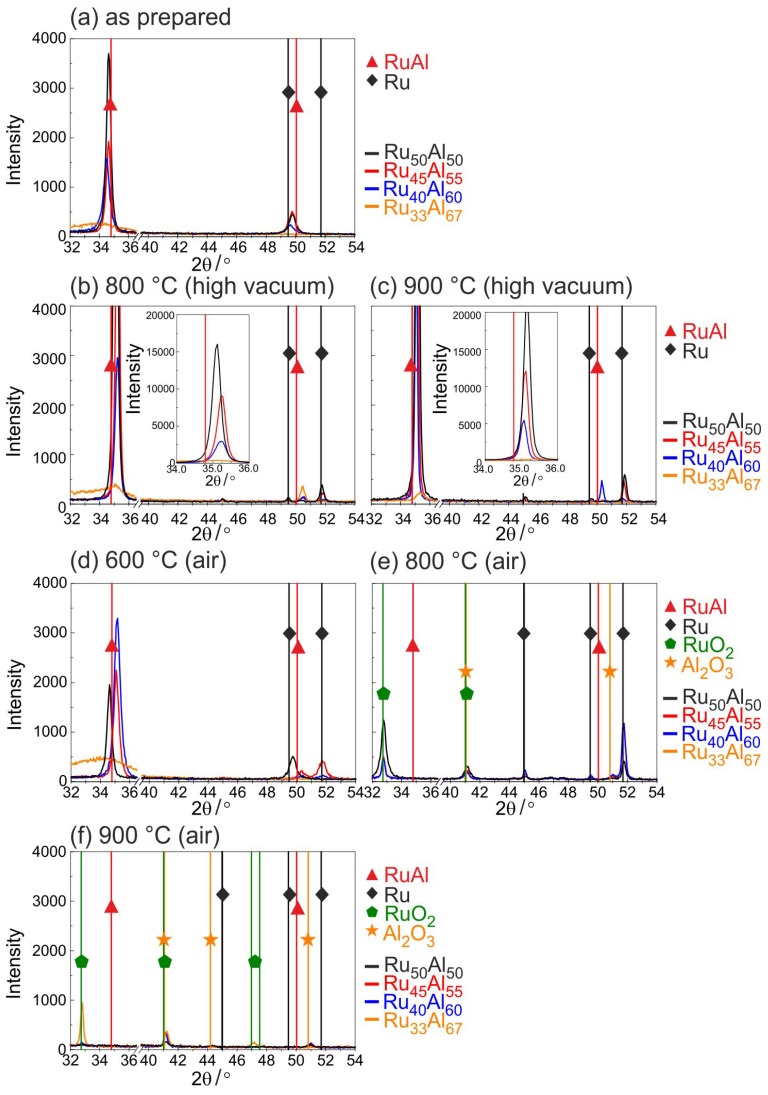
X-ray diffraction (XRD) measurements (CoK${}_{\alpha }$) of samples with the different compositions Ru${}_{100-x}$Al${}_{x}$ (x=50,55,60,67) (**a**) after preparation and after heat treatment for 10 h at (**b**) 800 ${}^{\circ }$C under high vacuum; (**c**) 900 ${}^{\circ }$C under high vacuum; (**d**) 600 ${}^{\circ }$C in air; (**e**) 800 ${}^{\circ }$C in air and (**f**) 900 ${}^{\circ }$C in air.

Figure 1 summarizes the results of the XRD measurements at different annealing temperatures between 600 and 900 ${}^{\circ }$C in air or under high vacuum conditions.

For the as-prepared state (Figure 1a), mainly (100) RuAl reflexes at a 2θ position of 34.8${}^{\circ }$ are visible. The highest intensity is reached for the Ru${}_{50}$Al${}_{50}$ sample. In contrast to this, the Ru${}_{33}$Al${}_{67}$ sample only shows a very small broad peak indicating amorphous phases. The RuAl (110) reflex is tiny for all samples and also not visible for the Ru${}_{33}$Al${}_{67}$ film.

Annealing under high vacuum conditions (Figure 1b,c) leads to a strong increase in the RuAl (100) intensity (a zoom-in of the RuAl (100) peak is shown in the inset). It can be seen that the reflex shifts to higher angles and that the intensity strongly depends on the composition. The lower the Al content, the higher is the intensity of the RuAl (100) reflex. Annealing at 900 ${}^{\circ }$C leads to higher RuAl (100) intensities than the annealing at 800 ${}^{\circ }$C. For the Ru${}_{33}$Al${}_{67}$ sample, only a very small RuAl (100) peak is visible for both temperatures. A small RuAl (110) reflex appears at 800 ${}^{\circ }$C for this sample, whereas at 900 ${}^{\circ }$C, this reflex is only visible for the Ru${}_{40}$Al${}_{60}$ film.

In addition to the RuAl reflexes, after the heat treatment, small Ru (101) reflexes also appear at a 2θ position of 51.7${}^{\circ }$ for the Ru${}_{50}$Al${}_{50}$ and Ru${}_{45}$Al${}_{55}$ samples.

Figure 1d–f presents the results of the XRD measurements after the heat treatment in air. Annealing at 600 ${}^{\circ }$C in air leads to a decrease of the RuAl (100) peak of the Ru${}_{50}$Al${}_{50}$ film. There is no change in intensity for the Ru${}_{45}$Al${}_{55}$ sample, but a clear increase of the peak intensity of the Ru${}_{40}$Al${}_{60}$ film is observed. In the case of Ru${}_{33}$Al${}_{67}$, there is a slight increase of the broad peak. An Ru (002) peak evolves for the Ru${}_{50}$Al${}_{50}$ sample and an Ru (101) peak for Ru${}_{45}$Al${}_{55}$.

The diffraction pattern changes clearly after the heat treatment at 800 ${}^{\circ }$C in air. The RuAl reflexes disappear for all samples and new phases appear. At 2θ of 32.4${}^{\circ }$, the RuO${}_{2}$ (110) reflex formed, which is strongest for the Ru${}_{50}$Al${}_{50}$ sample and nearly the same for the other samples. The small peak at 2θ of 41${}^{\circ }$ can be ascribed to RuO${}_{2}$ or Al${}_{2}$O${}_{3}$. A strong Ru reflex appears for Ru${}_{45}$Al${}_{55}$ and Ru${}_{40}$Al${}_{60}$, which is much smaller for the other two samples.

Finally, after the heat treatment at 900 ${}^{\circ }$C in air, only for the Ru${}_{33}$Al${}_{67}$ sample is an RuO${}_{2}$ (110) reflex present besides some very tiny RuO${}_{2}$/Al${}_{2}$O${}_{3}$ peaks.

### 3.2. Morphology

**Figure 2:**
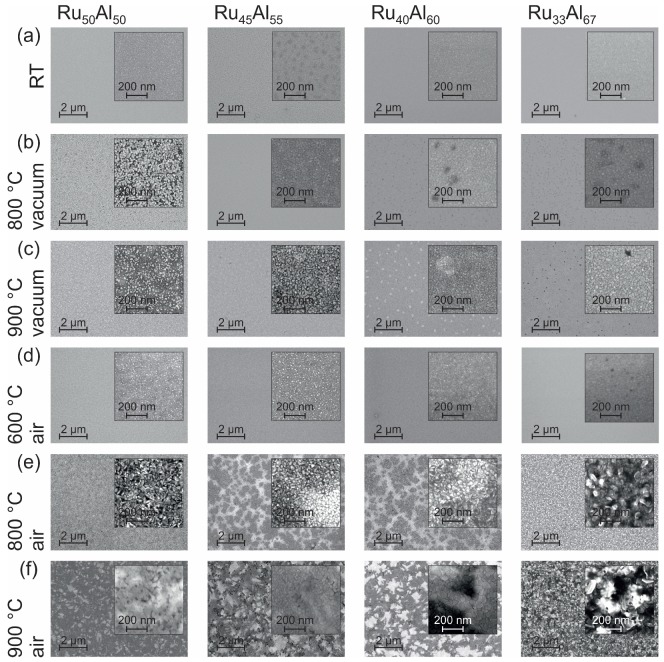
Scanning electron microscopy (SEM) inLens (20 kV) images showing the surface morphology of the samples with the different compositions Ru${}_{50}$Al${}_{50}$, Ru${}_{45}$Al${}_{55}$, Ru${}_{40}$Al${}_{60}$ and Ru${}_{33}$Al${}_{67}$ (**a**) after preparation and after heat treatment for 10 h at (**b**) 800 ${}^{\circ }$C; (**c**) 900 ${}^{\circ }$C under high vacuum, as well as (**d**) 600 ${}^{\circ }$C; (**e**) 800 ${}^{\circ }$C and (**f**) 900 ${}^{\circ }$C in air.

The surface morphology can be seen in the SEM images (inLens detector at 20 kV) summarized in Figure 2. At room temperature (RT) (Figure 2a), all samples appear homogeneous. Only in the case of Ru${}_{45}$Al${}_{55}$ is the surface a little bit spotted.

Annealing the samples at 800 ${}^{\circ }$C under high vacuum conditions (Figure 2b) only leads to minor changes. In the case of Ru${}_{50}$Al${}_{50}$, we find bright separated features in a darker matrix. For Ru${}_{40}$Al${}_{60}$ and Ru${}_{33}$Al${}_{67}$, darker areas are present.

The heat treatment at 900 ${}^{\circ }$C in high vacuum (Figure 2c) results in a further separation of the bright structures in the Ru${}_{50}$Al${}_{50}$ film and a starting separation of the features in the Ru${}_{45}$Al${}_{55}$ sample. Bright areas are formed in the Ru${}_{40}$Al${}_{60}$ film while the dark spots in Ru${}_{33}$Al${}_{67}$ become clearer.

The annealing procedure at 600 ${}^{\circ }$C under air conditions (Figure 2d) leads to the formation of very small bright spots at the sample surface. The density of those spots decreases with increasing Al content and becomes zero for the Ru${}_{33}$Al${}_{67}$ film.

The film morphology after the heat treatment at 800 ${}^{\circ }$C in air (Figure 2e) is completely different as compared to the previous samples. Larger structures with a high roughness have formed. In contrast to the films with a composition of Ru${}_{50}$Al${}_{50}$ and Ru${}_{33}$Al${}_{67}$, those with Ru${}_{45}$Al${}_{55}$ and Ru${}_{40}$Al${}_{60}$ possess large dark areas embedded in a brighter matrix.

The 900 ${}^{\circ }$C annealing under air (Figure 2f) shows a further modification of the morphology found for the 800 ${}^{\circ }$C air annealed samples. A patchy pattern is now also clearly visible for the Ru${}_{50}$Al${}_{50}$ sample. The surface appears smoother than after the 800 ${}^{\circ }$C air annealing. The samples Ru${}_{45}$Al${}_{55}$ and Ru${}_{40}$Al${}_{60}$ show also a pattern consisting of irregular bright and dark areas. Within these areas, there are also larger structures that appear very smooth. The most irregular surface structure with a strong contrast is found for the Ru${}_{33}$Al${}_{67}$ film.

### 3.3. Film Architecture

**Figure 3:**
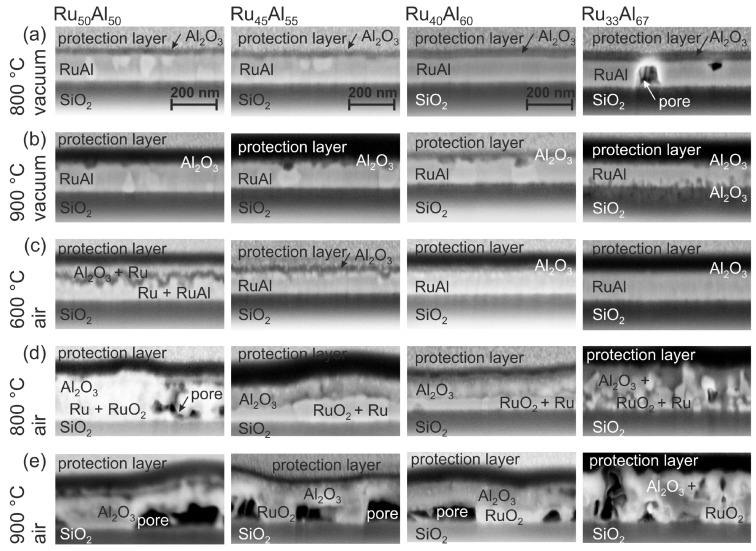
SEM inLens images (3 kV) of the focussed ion beam (FIB) cross sections of the samples with the different compositions Ru${}_{50}$Al${}_{50}$, Ru${}_{45}$Al${}_{55}$, Ru${}_{40}$Al${}_{60}$ and Ru${}_{33}$Al${}_{67}$ after heat treatment for 10 h at (**a**) 800 ${}^{\circ }$C; (**b**) 900 ${}^{\circ }$C under high vacuum, as well as (**c**) 600 ${}^{\circ }$C; (**d**) 800 ${}^{\circ }$C and (**e**) 900 ${}^{\circ }$C in air. The images are labeled according to the results of the Auger electron spectroscopy (AES) and transmission electron microscopy/energy dispersive X-ray spectroscopy (TEM/EDX) measurements.

SEM images of the cross sections of the films are presented in Figure 3. The labeling of the different layers was done according to the results of the AES and TEM/EDX measurements, which are discussed in the following sections. Clear differences between the various samples and annealing conditions are visible.

The cross section images of the samples annealed at 800 ${}^{\circ }$C under high vacuum (HV) conditions (Figure 3a) show randomly distributed bright grains in a matrix for the Ru${}_{50}$Al${}_{50}$ sample. The number of those grains is reduced for Ru${}_{45}$Al${}_{55}$. No grain structure is visible in case of Ru${}_{40}$Al${}_{60}$ and Ru${}_{33}$Al${}_{67}$; however, pores are formed in the latter film. These pores might be the origin of the dark spots that are visible in the SEM surface images.

After the heat treatment at 900 ${}^{\circ }$C under high vacuum (Figure 3b), still bright grains are present in the case of Ru${}_{50}$Al${}_{50}$ and Ru${}_{45}$Al${}_{55}$. A grain structure is now also visible in the Ru${}_{40}$Al${}_{60}$ sample. For these three films, small dark areas appear in the upper region of the film. In the case of the Ru${}_{33}$Al${}_{67}$ film, dark features have developed at the interface to the substrate. A cross section image of a more extended sample region of the Ru${}_{33}$Al${}_{67}$ film confirms pores also in this sample, which explain the dark spots in the surface images.

Annealing at 600 ${}^{\circ }$C under air conditions (Figure 3c) results in the case of Ru${}_{50}$Al${}_{50}$ and Ru${}_{45}$Al${}_{55}$ in a multilayer structure. There is a very irregular dark layer in the upper region of the film. This layer is much smoother in case of Ru${}_{45}$Al${}_{55}$. An additional thin bright layer is visible below the darker one. Such interlayers are not distinguishable in the other two systems. For Ru${}_{40}$Al${}_{60}$, the images reveal a thin layer of bright grains at the sample surface, while the sample with the highest Al content appears completely homogeneous.

The heat treatment at 800 ${}^{\circ }$C in air (Figure 3d) leads to pores in the lower region of the Ru${}_{50}$Al${}_{50}$ film. In contrast to this, the Ru${}_{45}$Al${}_{55}$ and Ru${}_{40}$Al${}_{60}$ samples are continuous with a two-layer structure. There are larger bright grains in the lower part of the film covered by a darker, more heterogeneous layer. The thickness of the lower layer changes laterally, which might also explain the differences in brightness in the SEM surface images. A completely inhomogeneous morphology has formed for the film with the Ru${}_{33}$Al${}_{67}$ composition. The large surface roughness that results from these grains confirms the roughness visible in the surface images.

The annealing process at 900 ${}^{\circ }$C in air (Figure 3e) results in the growth of the pores in the lower part of the Ru${}_{50}$Al${}_{50}$, Ru${}_{45}$Al${}_{55}$ and Ru${}_{40}$Al${}_{60}$ sample. These pores are covered by a continuous thick layer. The presence of these large pores also in this case explains the different structures, which are visible in the images taken of the sample surface. The Ru${}_{33}$Al${}_{67}$ sample behaves differently. In contrast to the other samples, this film is still quite compact with only small pores distributed randomly over the whole film thickness.

### 3.4. Film Chemistry

In the following, the results of the AES measurements concerning the film chemistry will be discussed. It has to be remarked that the AES results show mean values of the composition, since the layers are not homogeneous, e.g., Ru-rich grains and the RuAl-matrix as well as Al${}_{2}$O${}_{3}$ and Ru or RuO${}_{2}$ grains after annealing in air are measured simultaneously. Therefore, the AES results have to be interpreted with support of the XRD results and cross-section images.

**Figure 4:**
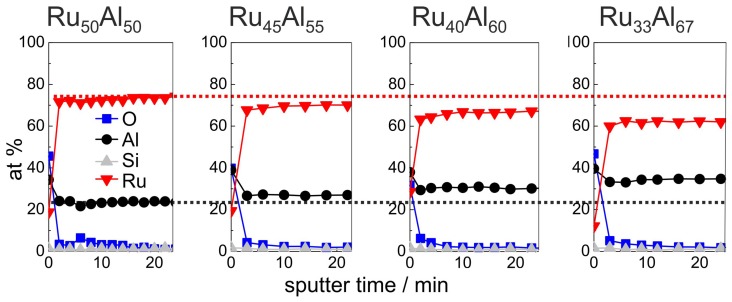
AES measurements of the as deposited samples with the different compositions Ru${}_{50}$Al${}_{50}$, Ru${}_{45}$Al${}_{55}$, Ru${}_{40}$Al${}_{60}$ and Ru${}_{33}$Al${}_{67}$. The red (black) dotted line marks the Ru (Al) content in the reference Ru${}_{50}$Al${}_{50}$ sample to visualize the difference to the other samples.

Figure 4 summarizes the initial 24 min of the AES measurements (for complete measurements, see Figure 7a) of the as-deposited samples, which verify the increasing Al and decreasing Ru content in the sample series, which was intended by the deposition. Additionally, the existence of a very thin Al${}_{2}$O${}_{3}$ oxide layer at the film surface is proven. However, the measured ratio between Ru and Al differs clearly from the deposited one, i.e., the absolute at % values that are derived from the AES measurements do not depict the actual composition. Further AES and XPS (X-ray photoelectron spectroscopy) investigations have shown that this is caused mainly by strong preferential sputtering effects in the Ru-Al-sytem [17], so that quantitative conclusions are not possible. However, the results allow a qualitative comparison between the samples.

**Figure 5:**
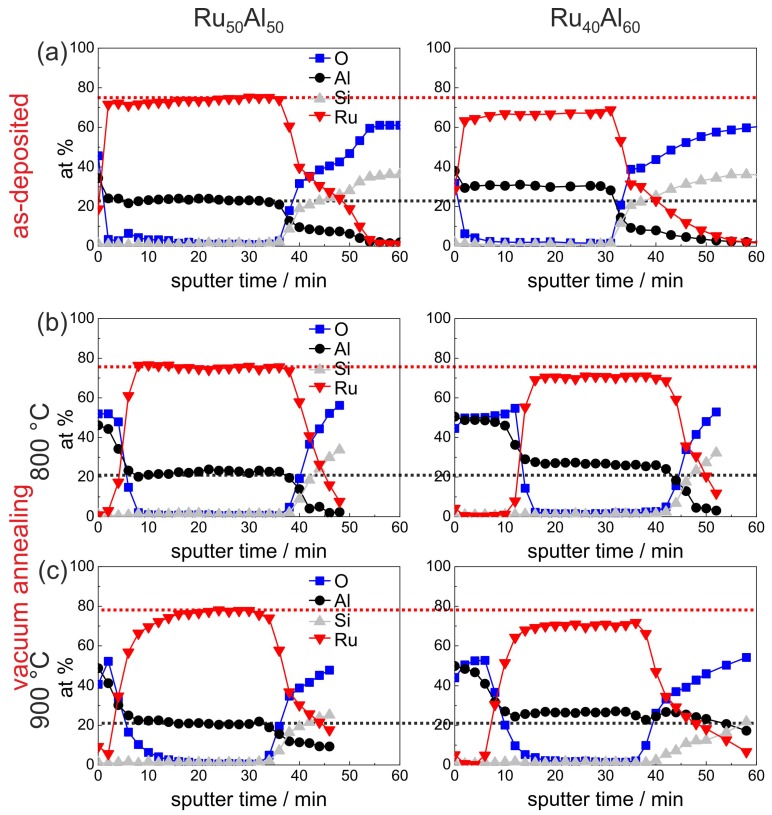
AES measurements of the Ru${}_{50}$Al${}_{50}$ and Ru${}_{40}$Al${}_{60}$ samples in (**a**) the as-deposited state and after annealing for 10 h in high vacuum at (**b**) 800 ${}^{\circ }$C and (**c**) 900 ${}^{\circ }$C. The red (black) dotted line marks the Ru (Al) content of the as-deposited reference Ru${}_{50}$Al${}_{50}$ sample to visualize the difference to the other samples.

The development of the sample chemistry after the various annealing procedures will be discussed based on the reference Ru${}_{50}$Al${}_{50}$ and exemplary Ru${}_{40}$Al${}_{60}$ samples. The overview image Figure 7, which summarizes the AES measurements of all samples and for all annealing procedures, shows that the behavior of the Ru${}_{45}$Al${}_{55}$ sample is between these two films. For the sample with the highest Al content, additional effects are visible which will be discussed subsequently. Figure 5 presents the measurements of the as-deposited and vacuum annealed state of the Ru${}_{50}$Al${}_{50}$ and Ru${}_{40}$Al${}_{60}$ samples. The results of the as-deposited films are repeated since they serve as a reference for the annealed samples.

Annealing at 800 ${}^{\circ }$C under HV conditions (Figure 5b) leads to the formation of an Al${}_{2}$O${}_{3}$ layer on top of the film. This layer is much thicker for the film with higher Al content (16 min of sputtering Al${}_{2}$O${}_{3}$ compared to 8 min). As a consequence of this oxide layer formation, the mean value of the measured Ru content increased slightly. In the case of the Ru${}_{50}$Al${}_{50}$ sample, the mean Ru content results from Ru-rich grains and a Ru-Al matrix (see Figure 3a), while such an inhomogeneity is not visible for the Ru${}_{40}$Al${}_{60}$ film.

Annealing at 900 ${}^{\circ }$C under HV conditions (Figure 5c) results in a broader transition between the Al${}_{2}$O${}_{3}$ layer on top of the film and the Ru-Al matrix in the case of the Ru${}_{50}$Al${}_{50}$ sample. In contrast to this, in the case of the Ru${}_{40}$Al${}_{60}$ film, the Al${}_{2}$O${}_{3}$ cover layer is thinner as compared to the 800 ${}^{\circ }$C case. Here, a different oxidation kinetics at 800 ${}^{\circ }$C as compared to 900 ${}^{\circ }$C has to be assumed, which leads to a more stable and more protective and with this thinner Al${}_{2}$O${}_{3}$ layer.

**Figure 6:**
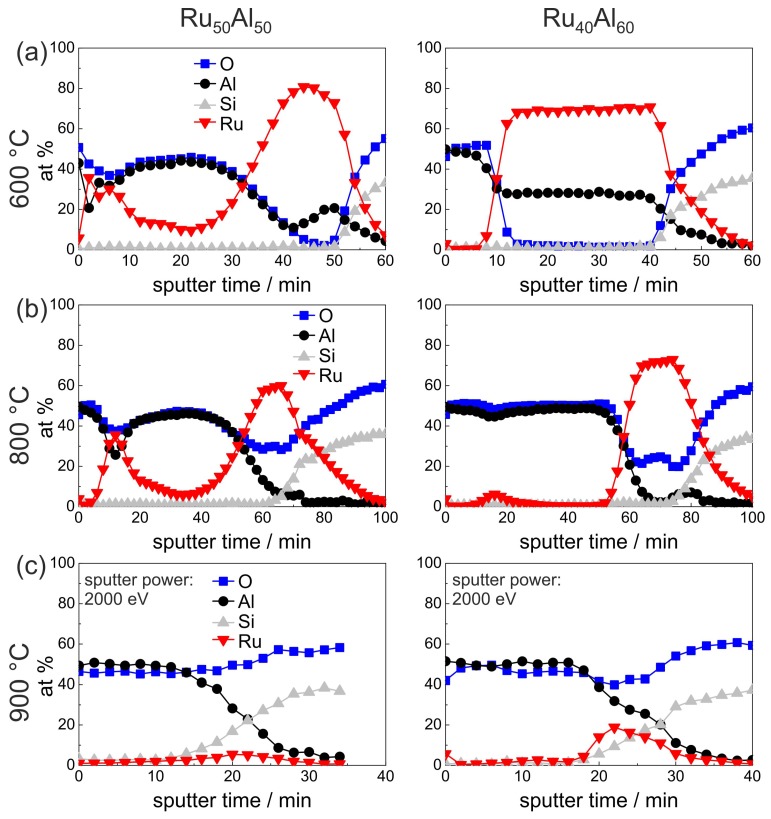
AES measurements of the Ru${}_{50}$Al${}_{50}$ and Ru${}_{40}$Al${}_{60}$ samples after annealing for 10 h in air at (**a**) 600 ${}^{\circ }$C; (**b**) 800 ${}^{\circ }$C; and (**c**) 900 ${}^{\circ }$C.

The AES results of the air-annealed Ru${}_{50}$Al${}_{50}$ and Ru${}_{40}$Al${}_{60}$ films are presented in Figure 6. A clear difference is visible after annealing at 600 ${}^{\circ }$C in air between both samples. While the Ru${}_{40}$Al${}_{60}$ film retains the bilayer Al${}_{2}$O${}_{3}$/Ru-Al/substrate architecture, the structure of the Ru${}_{50}$Al${}_{50}$ sample is completely different. Together with the cross section image (Figure 3c), the film architecture can be assumed as a multilayer structure. There is an Al${}_{2}$O${}_{3}$ layer at the surface, which also contains Ru. As can be seen from the cross section image, the layers on top of the film are very rough, so that, especially in this case, AES measures a mixture between the individual layers. Below the Al${}_{2}$O${}_{3}$, there is an Ru-rich and an Ru-Al layer, which are thinner than the remaining Ru-Al layer of the Ru${}_{40}$Al${}_{60}$ film. It has to be remarked that a quantitative relation between the sputter time and the film thickness of layers of different material is hardly possible due to the different sputter yields and the co-sputtering of several layers.

Also after annealing at 800 ${}^{\circ }$C in air, both samples differ strongly (Figure 6b). The sputter time necessary to reach the substrate has increased as compared to the measurements discussed above, especially for the Ru${}_{40}$Al${}_{60}$ film. The increase in sputter time can be attributed to the lower sputter yield of Al${}_{2}$O${}_{3}$ as compared to the metallic Ru-Al. The cross section images (Figure 3d) show a completely inhomogeneous film for the Ru${}_{50}$Al${}_{50}$ sample and the formation of pores near to the interface with the substrate. The AES measurements indicate the formation of a thick Al${}_{2}$O${}_{3}$ layer on top, which contains an accumulation of Ru near to the surface. Between the Al${}_{2}$O${}_{3}$ layer and the substrate there is a RuO${}_{2}$ layer. According to the XRD measurements (Figure 1), a small amount of metallic Ru is still present.

In contrast to this, the Ru${}_{40}$Al${}_{60}$ film has a much more homogeneous architecture with a thick Al${}_{2}$O${}_{3}$ layer on top, which does not contain any Ru besides a small accumulation at a sputter time of around 18 min. Below, there is a layer consisting of Ru and RuO${}_{2}$.

The measurement of the 900 ${}^{\circ }$C air annealed samples (Figure 6c) was performed with a higher ion beam energy, which was necessary due to charging of the sample surface due to the completely oxidic character of the remaining films. For the Ru${}_{50}$Al${}_{50}$ sample, the AES measurement showed hardly any remaining Ru within the film. A higher amount of Ru remains in the case of the Ru${}_{40}$Al${}_{60}$ sample, but the total amount of Ru has also decreased drastically. In both cases, the cross section images (Figure 3e) show the presence of large pores. However, for the Ru${}_{40}$Al${}_{60}$ film, RuO${}_{2}$ grains are also present. The decrease of the Ru content and the formation of cavities can both be ascribed to the further oxidation of RuO${}_{2}$ to gaseous RuO${}_{3}$ or RuO${}_{4}$ species.

**Figure 7:**
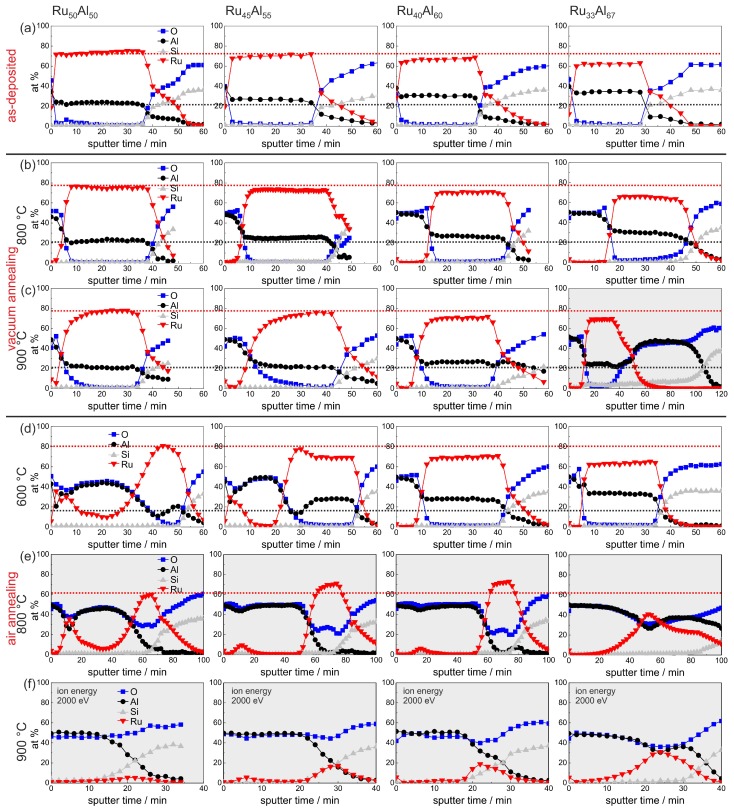
Overview summary of the AES measurements of the samples with the different compositions Ru${}_{50}$Al${}_{50}$, Ru${}_{45}$Al${}_{55}$, Ru${}_{40}$Al${}_{60}$ and Ru${}_{33}$Al${}_{67}$ (**a**) after preparation and after heat treatment for 10 h at (**b**) 800 ${}^{\circ }$C; (**c**) 900 ${}^{\circ }$C under high vacuum, as well as (**d**) 600 ${}^{\circ }$C; (**e**) 800 ${}^{\circ }$C; and (**f**) 900 ${}^{\circ }$C in air. The red (black) dotted lines mark the Ru (Al) content in the reference Ru${}_{50}$Al${}_{50}$ sample to visualize the difference to the other samples. The majority of the measurements is scaled for a sputtering time of 60 min, and the others are marked with the light gray background. Different sputtering conditions are remarked upon where they are applied.

An overview summary of the results of the AES measurements of all samples with the different compositions and for the different annealing temperatures is presented in Figure 7. In Figure 7a–e, the red and the black dotted lines indicate the maximum Ru and Al content, respectively, in the reference Ru${}_{50}$Al${}_{50}$ sample, which simplifies the comparison between the films. This assembly of all measurements, depending on both the initial composition and the annealing temperature, allows a direct comparison of all samples.

The sample with the lowest Ru content Ru${}_{33}$Al${}_{67}$ shows some peculiarities compared to the other films. The AES measurement of the 900 ${}^{\circ }$C vacuum annealed sample (Figure 7c) reveals a strong reaction between the film and the substrate. The sputtering of the Ru-Al layer is followed by a more than 40 min sputtering of an Al${}_{2}$O${}_{3}$ layer. However, the thickness of this layer is probably overestimated due to charging effects. A reaction between the film and the substrate after annealing at this temperature is also indicated by the cross section image (Figure 3b) where inhomogeneities between the Ru-Al layer and the substrate are visible. Moreover, after annealing at 900 ${}^{\circ }$C in air (Figure 7f), this sample contains the highest residual Ru content of all prepared samples in this series.

**Figure 8:**
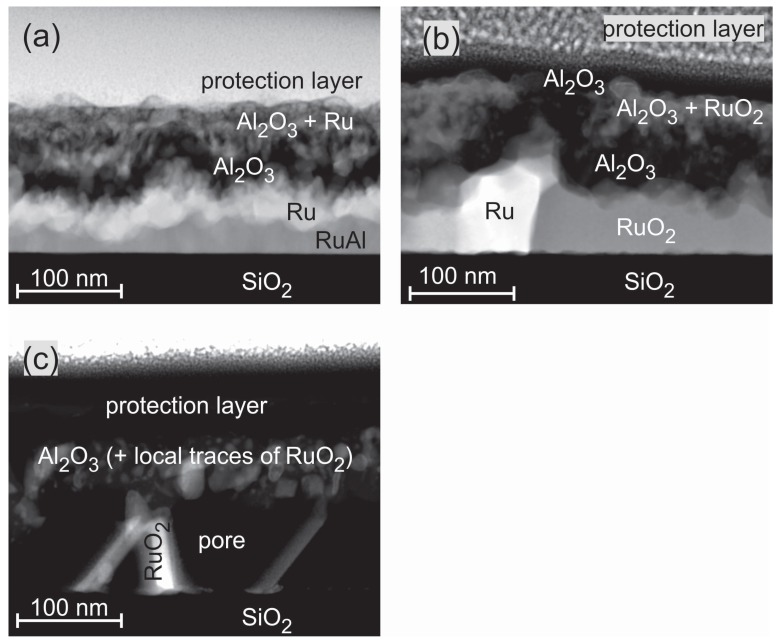
High-angle annular dark field scanning transmission electron microscopy (HAADF STEM) image (chemical contrast) of the Ru${}_{50}$Al${}_{50}$ sample annealed at (**a**) 600 ${}^{\circ }$C; (**b**) 800 ${}^{\circ }$C; and (**c**) 900 ${}^{\circ }$C for 10 h under air conditions.

Figure 8 presents HAADF STEM images of the exemplary Ru${}_{50}$Al${}_{50}$ samples for the annealing steps under air conditions. The images confirm the complicated film structure that was derived from the cross section SEM images and AES measurements. After annealing at 600 ${}^{\circ }$C, the STEM images show a four-layer structure (Figure 8a). Above the SiO${}_{2}$ substrate, a thin Ru-Al layer with varying thickness (≈10–50 nm) is present, followed by a layer consisting of pure Ru (≈40 nm). On top of it, there is a layer of pure Al${}_{2}$O${}_{3}$ (≈30 nm) followed by a matrix of Al${}_{2}$O${}_{3}$ with embedded Ru grains (≈20–50 nm). An Ru rich layer directly below the sample surface as indicated by the AES measurements was not confirmed in the particular position of the STEM measurement.

The annealing at 800 ${}^{\circ }$C (Figure 8b) under air conditions leads to the partial oxidation of Ru. Above the substrate, a layer consisting of Ru grains embedded in a RuO${}_{2}$ matrix has formed (≈40–90 nm). Above this layer, a thicker Al${}_{2}$O${}_{3}$ layer as compared to the 600 ${}^{\circ }$C case (≈60 nm) is present. For the 800 ${}^{\circ }$C sample, a thin layer (≈20 nm) with an accumulation of RuO${}_{2}$ grains within the Al${}_{2}$O${}_{3}$, which was detected by AES, is now clearly visible. The film is covered with a pure Al${}_{2}$O${}_{3}$ layer.

During the annealing of the sample at 900 ${}^{\circ }$C in air (Figure 8c), a further oxidation of the RuO${}_{2}$ takes place. The formation of gaseous RuO${}_{x}$ species leads to the development of pores in the former Ru/RuO${}_{2}$ layer. The upper part of the film consists of Al${}_{2}$O${}_{3}$. The EDX analysis proves the presence of few Ru species at single regions in the Al${}_{2}$O${}_{3}$. This fits with the AES data, which only show a small amount of Ru (up to 5 at %) close to the interface of the SiO${}_{2}$ substrate. The wide transition zone in the AES measurement between the film and the SiO${}_{2}$ evolves from large pores that account for more than 50 % of the lower layer between the scattered RuO${}_{2}$ grains. This leads to a simultaneous sputtering of RuO${}_{2}$ and the SiO${}_{2}$ substrate.

### 3.5. Electrical Properties

**Figure 9:**
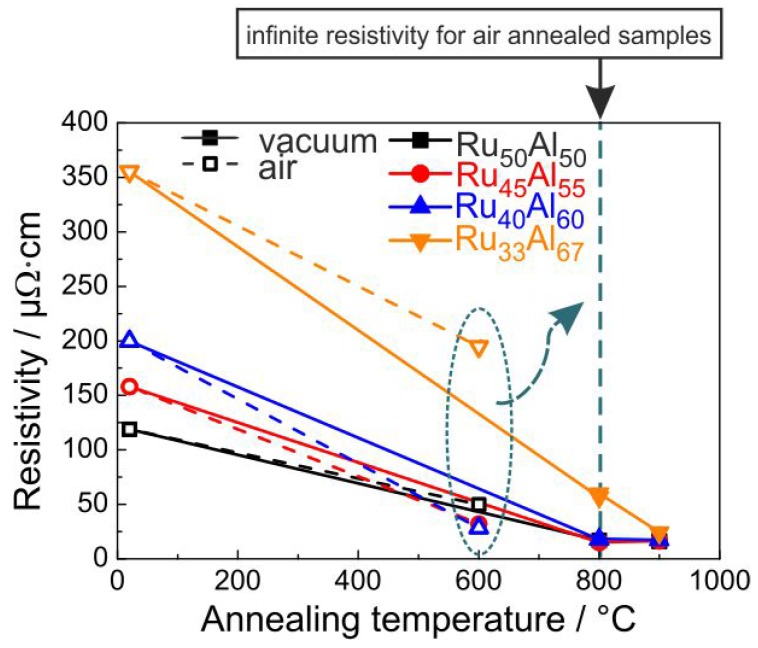
Electrical resistivity of the samples with the different compositions after annealing under high vacuum conditions (solid line) and air (dashed line); measured at RT. All films annealed in air at 800 ${}^{\circ }$C showed an infinite resistivity.

Since, for the application as metallization film, the electrical properties play an important role, the electrical resistivity was measured for the samples at RT after the annealing process. The results are presented in Figure 9. The resistivity was calculated from the sheet resistance considering the thickness of the films measured after deposition. Of course, due to the annealing process and the formation of the oxide layers, the real thickness of the conductive layer becomes smaller. The non-conductive oxides on top of the films and the formation of voids within the layers also complicate these measurements. Therefore, the results in Figure 9 represent only a mean value of the resistivity for the respective piece of sample.

It is obvious that a higher Al content leads to a higher resistivity for the as-prepared samples. The XRD measurements showed a reduced crystallization with increased Al content, which can explain this trend of the electrical properties. The heat treatment under high vacuum conditions leads to a strong reduction of the resistivity. After annealing at 800 ${}^{\circ }$C, all samples except the Ru${}_{33}$Al${}_{67}$ film (60 μΩ·cm) possess the same resistivity of about 17 μΩ·cm. The heat treatment at 900 ${}^{\circ }$C hardly changes the resistivity of the Ru${}_{50}$Al${}_{50}$, Ru${}_{45}$Al${}_{55}$ and Ru${}_{40}$Al${}_{60}$ samples. The value of Ru${}_{33}$Al${}_{67}$ further approaches the values of the other films and reaches 24 μΩ·cm. The clear reduction in electrical resistivity is in agreement with the crystal growth, which is visible from the sharper peaks and higher intensity in XRD measurements as seen in Figure 1. A better crystallization of the RuAl B2 phase with higher temperature also leads to fewer defects. Both effects additionally reduce the electrical resistivity.

The situation is different if the samples are annealed under air conditions. The resistivity values for the 600 ${}^{\circ }$C annealed samples are also presented in Figure 9. However, the measurements showed no conductivity for the samples annealed at higher temperatures. The 600 ${}^{\circ }$C annealing leads to the lowest resistivity for the Ru${}_{40}$Al${}_{60}$ sample (28 μΩ·cm), followed by the Ru${}_{45}$Al${}_{55}$ sample with a slightly higher value (32 μΩ·cm) and the Ru${}_{50}$Al${}_{50}$ sample with a clearly higher resistivity (50 μΩ·cm). Again, the highest value is observed for the Ru${}_{33}$Al${}_{67}$ film (195 μΩ·cm). The same qualitative behavior is observed for the RuAl peak heights in the XRD measurements, proving that there is a clear coherence between higher XRD peaks and a lower electrical resistivity.

All films annealed in air at 800 ${}^{\circ }$C showed an infinite resistivity. As bulk RuO${}_{2}$ has a relatively low electrical resistivity of 35 μΩ·cm [18], its formation does not explain why these samples are not electrically conductive anymore. Furthermore, FIB and STEM imaging of cross-sections showed a sparse distribution of pores well below the electrical percolation threshold.

Each electrical measurement was repeated four times and the direction of the electrical current was systematically changed, so that each measurement point consists of 16 individual measurements. The standard deviation of the measurements was in nearly all cases less than 0.1%, meaning that the respective error bar is smaller than the symbols shown in Figure 9. As mentioned above, the reduction of the effective thickness of the conductive layer is not considered in the calculation of the resistivity. Since the thickness of the conductive layer is smaller than the initial Ru-Al layer, the actual values of resistivity are smaller than the calculated ones.

## 4. Discussion

Several Ru-Al films with an intended Al content between 50 and 66 at % have been prepared and annealed at temperatures up to 900 ${}^{\circ }$C in air. However, different measurements revealed that the actual composition of the samples is shifted by 5 at % to the Ru-rich side. According to the Ru-Al phase diagram, there is an RuAl${}_{2}$ and Ru${}_{2}$Al${}_{3}$ phase [19]. Although the Al-rich Ru${}_{40}$Al${}_{60}$ and Ru${}_{33}$Al${}_{67}$ samples theoretically allow the formation of these phases, the XRD measurements only show RuAl reflexes in case of the Ru${}_{40}$Al${}_{60}$ film and an amorphous peak for Ru${}_{33}$Al${}_{67}$.

There is some literature concerning oxidation processes in Ru-Al systems. Mainly, these investigations consider bulk materials, produced by arc-melting or induction melting [13,14,20,21], ingot or powder metallurgy [12] or thick films (600 nm [10,22]), and there is only one source dealing with relatively thin films (160–300 nm [11]). The authors distinguish the behavior of single-phase RuAl samples and those with additional Ru or Ru/RuAl eutectic phases because the oxidation resistance strongly depends on the morphology of the samples.

In general, it is stated that the presence of a second phase besides RuAl, namely Ru, is detrimental for the formation of a protecting Al${}_{2}$O${}_{3}$ cover layer during annealing procedures. Bellina et al. state that Ru, which forms below the Al${}_{2}$O${}_{3}$ cover, hinders a further diffusion of Al towards the film surface [13]. Oxygen diffuses faster inside the sample than Al outwards, leading to the formation of another Al${}_{2}$O${}_{3}$ layer below the Ru rich layer [21]. For bulk materials, the authors describe the formation of a multilayer Al${}_{2}$O${}_{3}$/Ru or a porous Al${}_{2}$O${}_{3}$ multiscale layer if the oxidation temperature is sufficient for the formation of RuO${}_{2}$, and, subsequently, gaseous Ru oxides. The individual layer thicknesses of the Al${}_{2}$O${}_{3}$ or Ru/RuO${}_{2}$ layers is larger than the total film thickness of the samples investigated in this study. Therefore, such multilayer systems are not observable in our films.

However, the formation of an Al${}_{2}$O${}_{3}$/Ru bilayer is clearly visible from the cross section images (Figure 3c) for the Ru${}_{50}$Al${}_{50}$ and Ru${}_{45}$Al${}_{55}$ films after annealing at 600 ${}^{\circ }$C in air. The wavy interface between the Al${}_{2}$O${}_{3}$ and the Ru layer was similarly observed by Bellina et al. [13]. The Al${}_{2}$O${}_{3}$ cover is not protecting for these films, leading to an oxidation of Ru to RuO${}_{2}$ during annealing at 800 ${}^{\circ }$C (Figure 1e). Already at this temperature, the cross section image shows cavities for the Ru${}_{50}$Al${}_{50}$ film that arise from a further oxidation of RuO${}_{2}$ to gaseous RuO${}_{3}$ or RuO${}_{4}$. This effect also occurs for the Ru${}_{45}$Al${}_{55}$ film after annealing at 900 ${}^{\circ }$C.

Both films with higher Al content do not show a bilayer Al${}_{2}$O${}_{3}$/Ru structure after annealing at 600 ${}^{\circ }$C. It has to be assumed that these films contain sufficient Al to form an Al${}_{2}$O${}_{3}$ cover layer at this temperature and to maintain enough Al within the film to maintain a RuAl phase and to prevent the formation of a Ru-rich phase. Therefore, the Al${}_{2}$O${}_{3}$ cover is more protecting in these cases, which leads to a higher Ru content after annealing at 800 and 900 ${}^{\circ }$C in air. The lower the initial Ru content during deposition, the higher is the residual Ru content after the annealing (Figure 7f).

The growth rate of the Al${}_{2}$O${}_{3}$ scale at the surface of the sample was determined by Soldera et al. for melted RuAl samples with initial Ru${}_{50}$Al${}_{50}$ composition. These samples contain an Ru or Ru/RuAl-eutectic phase between the RuAl grains. The authors derived a linear time dependence of the thickening with about 1.75 μm/h [20]. On single phase RuAl bulk samples, a parabolic law for the growth of the oxide scale according to $x^{2}=kt+C$ with *k* being about 0.043 μm${}^{2}$/h and a *C* of 0.47 μm${}^{2}$ was concluded [14]. These growth rates were determined for an annealing at 1000 ${}^{\circ }$C in air and are much higher than the observed rates for the thin films investigated in this paper. Guitar et al. observed a higher oxidation resistance for thinner films (600 nm), since the higher density of grain boundaries facilitates a faster diffusion of Al to the surface [22]. This results in a faster formation of a dense Al${}_{2}$O${}_{3}$ scale, which is likely also the case for our samples.

## 5. Conclusions

A sample series of Ru-Al films with increasing Al content was investigated concerning the influence of the Al content on the phase formation and film stability after heat treatment either under vacuum or under air conditions. Although a higher Al content in the as-deposited films leads to a more homogeneous microstructure as seen from cross section images and AES measurements, the RuAl phase formation is not supported during annealing under high vacuum conditions. However, the additional Al leads to more stable RuAl films under air annealing at 600 ${}^{\circ }$C and to a higher residual Ru or RuO${}_{2}$ content after heat treatment even at higher temperatures in air.

The measurements also reveal that a higher Al content leads to a thicker Al${}_{2}$O${}_{3}$ cover layer after heat treatment, so that an estimation of the additional Al content, which is necessary for the RuAl phase formation during heat treatment, is hardly possible.

The results show that an excess of Al has a positive influence on the oxidation resistance but a negative effect on the RuAl phase formation. Therefore, a different approach has to be pursued to stabilize the RuAl phase in the Ru-Al films. Since the measurements also clearly prove that the Al${}_{2}$O${}_{3}$ layer that forms during the heat treatment does not really act as a passivating layer, additional covering layers are necessary to protect the Ru-Al layer against the environment and to stabilize the RuAl phase.
